# Supplementary material for: Predictors of homebirth amidst COVID-19 pandemic among women attending health facilities in Wondo Genet, Sidama Region, Ethiopia: A case control study
Source: PLoS One. 2023 May 2;18(5):e0283547. doi: 10.1371/journal.pone.0283547 (PMC10153687; doi:10.1371/journal.pone.0283547)
Supplement: S1 File — (DOCX) [file pone.0283547.s001.docx]

## Annex 1: Consent Form, English Version

**Information Sheet and Consent Form**

Hawassa University, College of Medicine and Health Sciences,

School of Public Health

Predictors of homebirth amidst COVID-19 pandemic among women attending health facilities in Wondo Genet, Sidama Region, Ethiopia: A Case Control

General Information for Study Participants

Good morning/afternoon.

My name is Mr. /Ms.……………………. I am postgraduate student of Hawassa University and I am doing research on determinants of home delivery in Wondo Genet. The results of this study could be used to improve maternal health services use in this town and surrounding community. I am going to ask you some questions regarding factors determining home delivery, and the interview takes 20 to 25 minutes. I would like to assure you that whatever information you provide will be kept strictly confidential and will not be shown to other people. In addition, your name will not be used in any document throughout this study. You have been selected at random to participate in this study. Your honest participation will help the researcher complete this study. There are no right and wrong answers; we need to know how you feel and why you made certain health-related decisions.

May I continue? Yes ……….. No ………..

If you say “yes” sign below

Date Consent form I have been briefly informed about the study and clearly understood the objective of the study. So I here approve my consent with my signature to take part in the study.

Signature ____________Date _____________

If no, please thank the interviewee and stop.

Interviewee’s signatures …………. Date………………….

Name of the principal Investigator: Asaminew Geremu

Mobile: 0954727451/52. E-mail: asaminewgaga1@gmail.com

Informed consent certified by:

Data collector code_______ Name __________________ Signature_________

Date of Interview___________ Time started __________Time completed ______

Results of interview: 1. Complete 2. Refused 3.Respondent not available 4.Partially completed.

Checked by: Supervisor Name __________ Signature ________Date _________

## Annex 1: English Version Questionnaire

Hawassa University, College of Medicine and Health Sciences, School of Public Health

Questionnaires to assess determinants of home delivery in Wondo Genet, South Ethiopia in 2021. 001. Questionnaire Code_______002.District/Town administration_____003.Kebele…………… 004. Name of health facility ______

005. Date of interview dd_____/mm_____/2021 006. Name of data collector ____________

007. Name of supervisor __________________ Check interview & sign here____________

008. Place of delivery 1-Home______ 2-Health facility __________3-Others________

| Part-1 Socio-demographic Characteristics of the Respondents | | | | |  |
| --- | --- | --- | --- | --- | --- |
| No | Question | Responses | | | R |
| 001 | How old are you? | ___________years | | |  |
| 002 | What is your level of education? | 1. No formal education 2. Primary(1-8) 3. Secondary(9-12) and above | | |  |
| 003 | What is your occupation? | 1. House wife 2. Merchant 3. Employer 4. Others_______________ | | |  |
| 004 | What is your marital status? | 1. Married 2. Single 3. Others* | | | If ‘2’ or ‘3’, skip to Q-007 |
| 005 | What is your husband’s level of education? | 1. No formal education 2. Primary(1-8) 3. Secondary(9-12) and above | | |  |
| 006 | What is occupation of your husband? | 1. Merchant 2. Farmer 3. Employer 4. Others^_______________ | | |  |
| 007 | Where is your place of residence? | 1. Urban 2. Rural | | |  |
| 008 | What is your religion? | 1. Protestant 2. Muslim 3. Orthodox 4. Others* | | |  |
| 009 | Which ethnic group do you belong? | 1. Sidama 2. Oromo 3. Amhara 4. Others** | | |  |
| 010 | What is your family’s average monthly income (in ETB)? | 1. <500 ETB 2. 500-1499 ETB 3. ≥ 1500 ETB | | |  |
| 011 | How frequently do you listen to/watch radio/TV? | 1. Never 2. Sometimes 3. Always | | |  |
| Part-2 Obstetric History Related Factors | | | | |  |
| 012 | How many times you had given live births? (parity) | | ……………live births | |  |
| 013 | Before your last pregnancy, have you used any contraceptive method? | | 1. Yes  2. No | |  |
| 014 | Was the pregnancy of last child wanted/planned | | 1. Yes  2. No | |  |
| 015 | Did you experienced difficult labor during delivery of last child? | | 1. Yes  2. No | |  |
| 016 | How many times did you receive ANC during your last pregnancy till delivery? | | 1. No visit at all  2. 1-3 time(s )  3. Four or more times | |  |
| 017 | How many months pregnant were you when you first received antenatal care for this pregnancy? | | 1. At 1st trimester  2. At 2nd trimester  3. At 3rd trimester | |  |
| 018 | Birth preparation  A. Did you save money?  B. Did you arrange transport?  C. Did you identified health institution and contacted health worker before delivery?  D. Did you identified a person who can donate blood in case need arises?  E. Did you have clean delivery kit? | | 1. Yes 2. No  1. Yes 2. No  1. Yes 2. No  1. Yes 2. No  1. Yes 2. No | |  |
|  | Did you prepared to before giving birth to your last child?* | | 1. Yes 2. No | |  |
| 019 | Where did you give birth for your youngest child? | | 1. At home 2. At health facility | | If 1, skip to Q-022 |
| 020 | If your answer for question 121 is at health facility, Why did you prefer it? | | 1. Difficult labor  2. Service is provided freely  3. Health facility was near to me  4. To get better or quality services  5. Other (specify)______ | |  |
| 021 | If you gave birth at home for your youngest child, Why do you prefer to deliver at home? (Multiple response is possible) | | 1. Labor was smooth /emergency  2.Lack of transport / ambulance  3. Not customary  4. Facility not open  5. Others (specify) ………. | |  |
| **Part-3 Knowledge of Obstetric Danger Signs** | | | | |  |
| 022 | In your opinion, what are some serious health problems that can occur during pregnancy that could endanger the life of a pregnant woman? | | 1. Vaginal bleeding 2. Fluid leakage before labor 3. Swelling of face/ extremities 4. Decreased/absent fetal movement 5. Other……………… 6. Don’t know…………… | |  |
| 023 | In your opinion, what are some serious health problems that can occur during labor and childbirth that could endanger the life of a pregnant woman? | | 1. Excessive vaginal bleeding 2. Placenta not delivered within 30 min after delivery 3. Convulsions 4. Prolonged labor( lasting >12 h) 5. Loss of consciousness 6. Other ……………… 7. Don’t know…………….. | |  |
| 024 | In your opinion, what are some serious health problems that can occur during the first 2 days after birth that could endanger the life of the woman? | | 1. Vaginal bleeding 2. Convulsion 3. Loss of consciousness 4. High fever 5. Malodourous vaginal discharge 6. Other (specify)………. 7. Don’t know……………… | |  |
| 025 | Now, I would like to ask you a few questions about the health of newborn babies. In your opinion, what are some serious health problems that can occur during the first 7 days after birth that could endanger the life of a newborn baby? | | 1. Difficulty/fast breathing 2. difficulty in suckling/feeding 3. bleeding or discharge from umbilical cord 4. yellowish discoloration of skin/eyes 5. Very small baby 6. Other (specify)……………… 7. Don’t know…………… | |  |
| **Part-3 Heath Service Related Factors** | | | | |  |
| 026 | Approximately how many hours does it take to reach nearby health facility? | | 1. <30 min 2. ≥30 min | |  |
| 027 | Perceived quality of services | |  | |  |
|  | a) When you go health facility for ANC or other services, how much time you stay until the end of the services? | | 1) <10min  2) 10-29min  3) >30min | |  |
|  | b) In any public health facility you ever visited for ANC or other services, did health professionals protect your privacy? | | 1) Yes  2. No  3. I don’t know | |  |
|  | c) According to your opinion, how friendly are the midwives in the health facility during ANC attendance or other services? | | 1) Friendly  2) Rude  3) I don’t know | |  |
|  | In your opinion, what did you perceive the quality of health services?* | | 1. Good 2. Poor 3. I don’t know | |  |
| 028 | Which gender of midwife would you prefer to assist you when you are giving birth? | | 1. Female 2. Male 3. I don’t mind any | |  |
|  | COVID-19 RELATED BARRIERS | |  | |  |
| 029 | During last pregnancy did you fear that COVID-19 may transmit to me or unborn baby if I go to health facility? | | 1. Yes 2. No | |  |
| 030 | During last pregnancy, have you experienced difficulty in accessing health services during COVID-19 due to lack of transport? | | 1. Yes 2. No | |  |
| 031 | During last pregnancy, have you experienced difficulty in accessing health services during COVID-19 due to lockdown? | | 1. Yes 2. No | |  |
| 032 | During last pregnancy, have you experienced difficulty in accessing health services during COVID-19 due to lack/non-use of mask | | 1. Yes 2. No | |  |
| Part-4 Factors Related to Women’s Autonomy and Domestic Violence | | | | |  |
| 033 | Women’s Autonomy | | |  |  |
|  | In your family, who is decider to get delivery services? | | | 1. Myself 2. We decide jointly 3. Husband only/others |  |
| 034 | **Lifetime Experience of Physical IPV** | | |  |  |
|  | In your lifetime, had your husband/intimate partner ever… | | |  |  |
|  | Slapped or had something thrown at you that could hurt you? | | | 1. Yes 2. No |  |
|  | Pushed or shoved you? | | | 1. Yes 2. No |  |
|  | Hit with fist or something else that could hurt you? | | | 1. Yes 2. No |  |
|  | Threatened to use or actually used a gun, knife, or other weapon against you? | | | 1. Yes 2. No |  |
|  | Choked or burnt on purpose? | | | 1. Yes 2. No |  |
|  | Kicked, drugged, or beaten up? | | | 1. Yes 2. No |  |
| 035 | **Lifetime Experience of Sexual IPV** | | |  |  |
|  | In your lifetime, had your husband/partner ever… | | |  |  |
|  | Physically force you to have sexual intercourse with him when you did not want to? | | | 1. Yes 2. No |  |
|  | Physically forced you to perform any other sexual act when you did not want? | | | 1. Yes 2. No |  |
|  | Threatened you to have sexual intercourse? | | | 1. Yes 2. No |  |
| 036 | **Lifetime Experience of Psychological IPV** | | |  |  |
|  | In your lifetime, had your husband/intimate partner ever… | | |  |  |
|  | Say or do something to humiliate you in front of others? | | | 1. Yes 2. No |  |
|  | Threaten to hurt or harm you or someone you cared about? | | | 1. Yes 2. No |  |
|  | Insult you or make to feel bad about yourself? | | | 1. Yes 2. No |  |
|  | Done things that scare or intimidate you? | | | 1. Yes 2. No |  |
| 037 | **Experience of Physical IPV during pregnancy** | | |  |  |
|  | During your last pregnancy, had your husband/partner ever… | | |  |  |
|  | Slapped or had something thrown at you that could hurt you? | | | 1. Yes 2. No |  |
|  | Pushed or shoved you? | | | 1. Yes 2. No |  |
|  | Hit with fist or something else that could hurt you? | | | 1. Yes 2. No |  |
|  | Threatened to use or actually used a gun, knife, or other weapon against you? | | | 1. Yes 2. No |  |
|  | Choked or burnt on purpose? | | | 1. Yes 2. No |  |
|  | Kicked, drugged, or beaten up? | | | 1. Yes 2. No |  |
| 038 | **Experience of Sexual IPV during pregnancy** | | |  |  |
|  | During your last pregnancy, had your husband/partner ever… | | |  |  |
|  | Physically force you to have sexual intercourse with him when you did not want to? | | | 1. Yes 2. No |  |
|  | Physically forced you to perform any other sexual act when you did not want? | | | 1. Yes 2. No |  |
|  | Threatened you to have sexual intercourse? | | | 1. Yes 2. No |  |
| 039 | **Experience of Psychological IPV during pregnancy** | | |  |  |
|  | During your last pregnancy, had your husband/partner ever… | | |  |  |
|  | Say or do something to humiliate you in front of others? | | | 1. Yes 2. No |  |
|  | Threaten to hurt or harm you or someone you cared about? | | | 1. Yes 2. No |  |
|  | Insult you or make to feel bad about yourself? | | | 1. Yes 2. No |  |
|  | Done things that scare or intimidate you? | | | 1. Yes 2. No |  |

*to be filled by the interviewer
