# Supplementary material for: Predictors of homebirth amidst COVID-19 pandemic among women attending health facilities in Wondo Genet, Sidama Region, Ethiopia: A case control study
Source: PLoS One. 2023 May 2;18(5):e0283547. doi: 10.1371/journal.pone.0283547 (PMC10153687; doi:10.1371/journal.pone.0283547)
Supplement: S2 File — (DOCX) [file pone.0283547.s002.docx]

## Annex 2: Consent Form, Sidaamu Afoo Version

Mashalaqqete Woraqatanna Sumiimmate Forme

Hawaasi Yuniversite Meedisiinetenna Fayyimmate Saayinse Kolleejje

Dagate Fayyimma Rosi Mine

Godowinni Noo Amuwira Mine Ilate Korkaata Ikkannore Sidaamu Dagoomu Qoqqowu Mangiste, Wondo

Genetete, 2013 M.D.

Xa’mamaanchchonnita Wo’ma Mashalaqqe

Keere galitta/hositta?

Su’ma’ya Kalaa/Baabba……………………………………………………………………. Yaamameemmo/mma.Ani hawaasi Yuniversiite rosaanchooti. Wondogenetete giddo iltanno amara mine ilate korkaata ikkannorichchi aana buuxo assanni noommo. Tenne buuxote gumi Wondo Genetete giddo ikko ollaaho ama mine iltannokki gedenna fayyimmate uurrinsha giddo iltanno gede kaa’lo ikkitanno. Konni daafira lemuunni lemiina ontu xiqqessi geeshsha adhitanno yanna giddo xa’mo xa’meemmohe.Xa’meemmohe xa’mora qolatta qolo ayeerano dikullanni ledoteno su’makki diborreessinani. Tenne buuxora ate hedeweelchcho doorroonni. Danchchu garinni xa’mo qolittaero tenne buuxo injino garinni gudate kaa’lanno.Xa’mo xa’mineenna qolatta qolora addaho woyi xaraho yinanni qolo dino. Hasi’nannihulla kaayinni mine woyi fayyimmate agarooshshira ilate korkaata ikkinoherichcho afateeti.

Xa’ma dandeemmo? Ee ……….. Dee’ni ………..

Sumuu yoottaro, woroonni malaatekki wori

Date……………………………Consent form …………………

Ani tenne buuxo korkaatanna mashalaqqe wo’munni wo’ma afoomma.Konni daafira woroonni malaatisatenni tenne buuxora xa’mo qolatenni sumiimma’ya xawiseemma.

Malaate ____________Barra _____________

Sumuu yitannokkiro, galaxxeemmohe yite aguri.

Xa’mamaanchchote malaate………………… Barra………………………………………….

Marrajja Gamba assannohu su’ma…………………………………..Malaate……………………………….

Buuxaanchchu Su’ma: Asaminew Geremu

Mobaayile: 0954727451/52. Emeile: asaminewgaga1@gmail.com

Sumiimmate forme buuxxinori:

Mashalaqqe gamba assannohu koodde……………….. Su’ma __________________ Malaate_________

Xa’minoonni barra___________ Hananfoonni yanna __________Gundoonni yanna ______

Xa’mote gumulo: 1. Wo’ma 2. Gibbino 3.Xa’mo qoltannoti dino 4.Gama wo’mitino.

Buuxinohu: Supervayzerete su’ma __________ Malaate ________Barra _______

## Annex 2: Sidaamu Afoo Version Questionnaire

**Xa’mote Woraqata Sidaamu Afiinni**

001. Xa’mote woraqati koodde________ 002. Woradu/Kuchchumu gashshoote______003.ollaa…………… 004. Fayyimmate agarooshshi su’ma ____ 005. Xa’minoonni barra: Barra_____/Agana_____/2021 006. Xa’mannohu su’ma ________007.Superivayizerete su’ma ___Xa’mo buuxxe kowe malaatisi____008. Iltino bayiichcho 1-Mine______ 2-Fayyimmate agarooshshi giddo ______3-Wole darga________

| Part-1 Xa’mamaanchchonnita Daganna Dagoomittete Akata | | | | | | |
| --- | --- | --- | --- | --- | --- | --- |
| Kiiro | Xa’mo | | Dawaro | | | La’ooshe |
| 001 | Dirikki me’eho? | | ……………………….dirooti | | |  |
| 002 | Roso me’e kifle geeshsha rosootta? | | 1. Horontanni dirosoomma 2. Umi dirima(1-8) 3. Layiinkkinna dirimma (9-12) nna aleenni | | |  |
| 003 | Mini looso agurranna womaashshu woyi uduunnichu e’o afiratta looso maa loosatta? | | 1. Dilooseemma 2. Daddalaanchchote 3. Mangistete loosaasinchchooti 4. Wole^_______________ | | |  |
| 004 | Adhamate gari hiittooho? | | 1. Adhamoomma 2. Diadhamoomma 3. Woloota** | | | 2 woy 3 ikkiro, 007 sa’i |
| 005 | Maatekki me’e kifle geeshsha rosino? | | 1. Horontanni dirosino 2. Umi dirima(1-8) 3. Layiinkkinna dirimma (9-12) nna aleenni | | |  |
| 006 | Minikki maate loosi maati? | | 1. Daddalaanchchoho 2. Baatto loosire galinoho 3. Mangistete looosaasinchchooti 4. Wole^_______________ | | |  |
| 007 | Xa heeratta dargi mamaati? | | 1. Quchchumu giddo 2. Baddeeyyete | | |  |
| 008 | Amma’nokki maati? | | 1. Pirotesitaantete 2. Musiliimete 3. Oritodokisete 4. Woloota* | | |  |
| 009 | Hiittenne daga giddo gaamamatta? | | 1. Siidaamaho 2. Oromote 3. Amaaraho 4. Woloota | | |  |
| 010 | Mini’ne giddo aganunni afidhinanni eo itiyopiyu birrenni me’ete? | | 1. <500 birreeti 2. 500-1499 birreeti 3. ≥ 1500 birreeti | | |  |
| 011 | Televizhiine woyi Radoone me’e higge la’aa? | | 1. Dee’ni 2. Sa’e sa’e 3. Wo’ma woyiite | | |  |
| 012 | Xaa geeshsha me’e higge iloo? | | ………………hige | | |  |
| 013 | Konne qaaqqo godowakkira albaanni, ila xeertinsannirichcho horonsidhe egennoo? | | 1.ee 2.dee’ni | | |  |
| 014 | Konne xa kittiwaateho abbirootta qaaqqo godowitta waro hasidhdheetinso hasirittakkinniiti godowaminoho? | | 1.ee 2.dee’ni | | |  |
| 015 | Xaa geeshsha iltanni qarrante egennootta? | | 1.ee 2.dee’ni | | |  |
| 016 | Konne qaaqqo godobbe heedhe kittiwaate qasirate fayyimmate uurinsha me’e higge harootta? | | 1. Diqasiroomma 2. 1-3 geeshsha 3. 4 nna roore hige | | |  |
| 017 | Umi kittiwaate qasiritta waro godowikkira me’e aganaati? | | ………………………….aganaati | | |  |
| 018 | Konne qaaqqo ilakkira albaanni qixxawo assootta?   1. Birre codhootta? 2. Hodhishsha qixxeessirootta? 3. Ilattaha fayyimmate agarooshshi uurrinshanna oggeeyye afirootta? 4. Hasiissuha ikkiro mundee aannohe manna qixxeessirotta, 5. Ilate ikkanno uduunnichcho qixxeessirootta? | | 1. codhoomma 2. Dicodhoomma  1. qixxeessiroomma 2. diqixxeessiroomma   1. qixxeessiroomma 2. Diqixxeessiroomma   1. qixxeessiroomma 2. diqixxeessiroomma  1. qixxeessiroomma 2. Diqixxeessiroomma | | |  |
| 019 | Konne qaaqqo hiikko iloo? | | 1. Mine/qachchaho  2. Fayyimmate uurrinsha giddo | | |  |
| 020 | Fayyimmate uurrinshara iloottaro, fayyimmate uurrinsha doodhakkira korkaatu maati? | | 1. Gamaa’mate aana qarrame  2. Baatooshshiweelo owaante uyinanni  3. Fayyimmate uurrinsha mule ikkitino daafira  4. Woyyaabbino owaante afirate  5. Wole (haransite)_______ | | |  |
| 021 | Konne qaaqqo mine iloottaro, mine ilate korkaata ikkihehu maati? | | 1. Game hedeweelchcho hanaffinoe  2. Hodhishsha hooge  3. Fayyimate uurrinshara ila hasiissannokki daafira  4. Fayyimmate uurrinsha cufote  5.wole(haransite) | | |  |
| Part-3 **Amatenna Qaaqqu Aana Leellanoha Jaddote malaate afate xa’mo** | | | | | |  |
| 022 | Umikki hedonni, godowinni hee’neenna leellara dandaannoha jaddote malaate maatiro afootta/maati? | 1. Mundee du’nama 2. Game hanaffukkinni wayi gedeerichchi fula 3. Lowo geeshsha iibbabbisha 4. Qaaqquullu godo’la aja/hooga 5. Wole…………………….. 6. Diafoomma………………………….. | | | |  |
| 023 | Umikki hedonni, gamaa’minanninna illanni woyiite iillara dandaannoha jaddote malaate maatiro afootta/maati? | 1. Lowo mundee du’nama 2. Hobbaate sajjuu xiqqeessi giddo ilama hooga 3. Huxisa 4. Seeda yanna gamaa’minanni keeshsha (>12 saate) 5. Umo hawa 6. wole ……………….. 7. Diafoomma…………………….. | | | |  |
| 024 | Umikki hedonni, qaaqqo ille ka’neenna, lamu barri giddo amate lubbo gawajjote giddora eessa dandaanno malaate afootta/maati? | 1. Lowo mundee du’nama 2. Huxisa 3. Lowo geeshsha iibbabisha 4. Bowannorichchi fula 5. Umo hawa/daafursa 6. wole ……………….. 7. Diafoomma | | | |  |
| 025 | Xa qaaqqu aana leellannorichcho xa’meemmohe. Umikki hedonni, qaaqquullu ilame kaeenna lamalu barri giddo leellara dandaannohu jaddote malaati maati? | 1. Foo’la hooga/foo’late qarrama 2. Unuuna qana hooga 3. Suuwote giddonni mundee fula 4. Illete woyi bisoho bakkala malaate leellisha 5. Lowo geeshsha shiima/qelpheepho aja 6. Wole…………… 7. Diafoomma………… | | | |  |
| **Parte-3 Fayyimate Owaante Lainohunni** | | | | | |  |
| 026 | Minikkinni fayyimmate uurrinsha iillate me’e yanna adhanno? | | | 1) 30 xiqqeessi woroonni  2) 30 xiqqeessi aleenni | |  |
| 027 | Owaantete Isilanchchimma | | |  | |  |
|  | a) Fayyimmate xaawira, owaante afidhe ka’atta geeshsha mageeshshi yanna adhano? | | | 1) <10’  2) 10-29’  3) >30’ | |  |
|  | b) fayyimmate xaawira fojokki agarranni gari hiittooho? | | | 1.Danchchaho  2. Bushaho/diafoomma | |  |
|  | c) Fayyimmate xaawi loosaasine, ate danchchu garinni shiqqe loossanno? | | | 1.Ee  2. Dee’ni/diafoomma | |  |
| 028 | Qaaqqo ilatta woyiite hiikku kaa’lahera hasiratta? | | | 1. Meyaati 2. Labbaahu 3. Ayeeno ikkiro qarru dino | |  |
|  | **Koroonu Vaayirese Lainohunni** | | |  | |  |
| 029 | Konne qaaqqo godobbe heedhe, fayyimmate xaawira harummaro koroonu xisso anera woyi qaaqqoho sa’aera dandiitanno yite waajjite egennootta? | | | 1. ee 2. dee’ni | |  |
| 030 | Konne qaaqqo godobbe heedhdhe, koroonu korkaatinni fayyimmate xaawa haratenni hodhishsha hoogge gatte egennoo? | | | 1. ee 2. dee’ni | |  |
| 031 | Konne qaaqqo godobbe heedhdhe, koroonu korkaatinni fayyimmate xaawa haratenni mininni fulate shette gatte egennoo? | | | 1. ee 2. dee’ni | |  |
| 032 | Konne qaaqqo godobbe heedhdhe, koroonu korkaatinni fayyimmate xaawa haratenni maaske woyi sanitaayizere hoogge gatte egennoo? | | | 1. ee 2. dee’ni | |  |
| 033 | **Amate wossanate wolqa** | | | |  |  |
|  | Mini’ne giddo, godowinni heedhe kitiwaate qasirate woyi ilate la’annosihu ayeeti? | | | | 1-Aneeti  2-Lamunku mitteenni  3-Minaanni/wolu |  |
| 034 | **Heeshshote Diri Giddo Iillitinota Mannimmate Aani Jaddo** | | | |  |  |
|  | Heeshsho’ne diri giddo, minaannikki………….. | | | |  |  |
|  | Qawaade woyi mitirichchinni ole gane egenneewohe? | | | | 1. ee 2. dee’ni |  |
|  | Xiiwe tuge egennewoohe? | | | | 1. ee 2. dee’ni |  |
|  | Tuntu’motenni jane egennewohe? | | | | 1. ee 2. dee’ni |  |
|  | Qaraamurichchinni jane woyi qase egenneewohe? | | | | 1. ee 2. dee’ni |  |
|  | Qalxe woyi afayiheereyi giire egennewohe? | | | | 1. ee 2. dee’ni |  |
|  | Lekkatenni kade, woyi uullulate aana goshooshe egennewohe? | | | | 1. ee 2. dee’ni |  |
| 035 | **Heeshshote Diri Giddo Iillitinota Xadooshshu Gawajjo** | | | |  |  |
|  | Heeshsho’ne diri giddo, minaannikki……….. | | | |  |  |
|  | Ati hasirittakkinni ugge xaadooshshe asse egenninohe? | | | | 1. ee 2. dee’ni |  |
|  | Ati hasiroottakki garinni xadooshshe asse egenninohe? | | | | 1. ee 2. dee’ni |  |
|  | Xadooshshe assara addi addi garinni waajjishiishe egenninohe? | | | | 1. ee 2. dee’ni |  |
| 036 | **Heeshshote Diri Giddo Iillitinota Dadillisate gawajjo** | | | |  |  |
|  | Heeshsho’ne diri giddo, minaannikki……… | | | | 1. ee 2. dee’ni |  |
|  | Mannu albaanni saalfachchishe egenneewohe? | | | | 1. ee 2. dee’ni |  |
|  | Atenna ate ledo noo manna waajjishiishenna hanqe egenneewo? | | | | 1. ee 2. dee’ni |  |
|  | Xone woyi koffi asse egenninohe? | | | | 1. ee 2. dee’ni |  |
|  | Jajjarse woyi waajjishiishe egennewohe? | | | | 1. ee 2. dee’ni |  |
| 037 | **Godowinni heedheenna Iillitinota Mannimmate Aani Jaddo** | | | |  |  |
|  | Godowinni heedheenna, minaannikki………….. | | | |  |  |
|  | Qawaade woyi mitirichchinni ole gane egenneewohe? | | | | 1.ee 2. dee’ni |  |
|  | Xiiwe tuge egennewoohe? | | | | 1.ee 2. dee’ni |  |
|  | Tuntu’motenni jane egennewohe? | | | | 1.ee 2. dee’ni |  |
|  | Qaraamurichchinni jane woyi qase egenneewohe? | | | | 1.ee 2. dee’ni |  |
|  | Qalxe woyi afayiheereyi giire egennewohe? | | | | 1.ee 2. dee’ni |  |
|  | Lekkatenni kade, woyi uullulate aana goshooshe egennewohe? | | | | 1.ee 2. dee’ni |  |
| 038 | **Godowinni heedheenna Iillitinota Xadooshshu Gawajjo** | | | |  |  |
|  | Godowinni heedheenna, minaannikki……….. | | | |  |  |
|  | Ati hasirittakkinni ugge xaadooshshe asse egenninohe? | | | | 1.ee 2. dee’ni |  |
|  | Ati hasiroottakki garinni xadooshshe asse egenninohe? | | | | 1.ee 2. dee’ni |  |
|  | Xadooshshe assara addi addi garinni waajjishiishe egenninohe? | | | | 1.ee 2. dee’ni |  |
| 039 | **Godowinni heedheenna Iillitinota Dadillisate gawajjo** | | | |  |  |
|  | Godowinni heedheenna minaannikki……… | | | | 1.ee 2. dee’ni |  |
|  | Mannu albaanni saalfachchishe egenneewohe? | | | | 1.ee 2.dee’ni |  |
|  | Atenna ate ledo noo manna waajjishiishenna hanqe egenninino? | | | | 1.ee 2. dee’ni |  |
|  | Xone woyi koffi asse egenninohe? | | | | 1.ee 2. dee’ni |  |
|  | Jajjarse woyi waajjishiishe egennewohe? | | | | 1. ee 2. dee’ni |  |

*konne xa’mannohu wonshanno
