# Supplementary material for: Predictors of homebirth amidst COVID-19 pandemic among women attending health facilities in Wondo Genet, Sidama Region, Ethiopia: A case control study
Source: PLoS One. 2023 May 2;18(5):e0283547. doi: 10.1371/journal.pone.0283547 (PMC10153687; doi:10.1371/journal.pone.0283547)
Supplement: Attachment — Submitted filename: Response to Reviewers.docx [file pone.0283547.s004.docx]

Yilkal Simachew

Lecturer and Researcher

Hawassa University, Ethiopia

[joemakalister123@gmail.com](mailto:joemakalister123@gmail.com)

Dr. Shabnam Iezadi

Academic Editor

*Journal of PLOS ONE*

[em@editorialmanager.com](mailto:em@editorialmanager.com)	January, 03, 2023

**Subject:** Revision and resubmission of manuscript (PONE-D-22-17306)

Dear Dr. Shabnam Iezadi

Thank you for giving us the opportunity to submit a revised draft of the manuscript “Predictors of homebirth amidst COVID-19 pandemic among women attending health facilities in Wondo Genet, Sidama Region, Ethiopia: a case control study”, for publication in the *journal of PLOS ONE*. We appreciate the time and effort that you and the reviewers dedicated to providing feedback on our manuscript and grateful for the insightful comments on and valuable improvements to our paper. We have incorporated most of the suggestions made by the reviewers. The changes are marked in the revised manuscript.

We have included the Reviewers and Editor Comments immediately after this letter and responded to them individually, indicating exactly how we addressed each concern and describing the changes we have made. Please note that reviewers’ comments are shown in **bold** type and our responses in plain type. Changes to the manuscript that have been made are marked in blue color in the revised version. The removal of text at certain locations is highlighted with ~~red~~ color in the revised manuscript.

We hope the revised manuscript will better suit the *journal of PLOS ONE,* and we thank you for your continued interest in our research.

Sincerely,

Yilkal Simachew

Lecturer and Researcher at Hawassa University, in Ethiopia

**REPLY TO ACADEMIC EDITOR’S COMMENTS**

**1. Please ensure that your manuscript meets PLOS ONE's style requirements, including those for file naming. The PLOS ONE style templates can be found at**

**Response:** Checked and corrected; we have now reformatted the manuscript (font size, font style, line spacing, figure caption, table caption, reference citation and file naming) according to the guidelines and style requirements of PLOSE ONE.

**2. You indicated that you had ethical approval for your study. In your Methods section, please ensure you have also stated whether you obtained consent from parents or guardians of the minors included in the study or whether the research ethics committee or IRB specifically waived the need for their consent.**

**Response:** Our study subjects were mothers who gave birth in the last 12months prior to the study. The nine (2.9%) mothers less than 18 years old who were participated in this study were already married and do not live with the parents. Verbal consent to participate in the study was obtained from these mothers (3 cases and 6 controls) and approved by the IRB. The IRB is well aware that the respondents in this study were married and already left their parents. If a woman marries before the age of 18 years old, she leaves her parents and lives her life independently of her parents. The IRB is aware that this is a reality in Ethiopia and thus, it approved the consent without requiring additional parental consent. Based on the given comments, now we have stated this on method section (Revised manuscript, line #298-301).

**3. In the ethics statement in the Methods, you have specified that verbal consent was obtained. Please provide additional details regarding how this consent was documented and witnessed, and state whether this was approved by the IRB**

**Response:** Based on the suggestions, we clarify how the verbal consent was documented and witnessed, and statement about the IRB approval *“The data collector read the information sheet and consent to the participants slowly and loudly. Then they were asked if they had any questions. After the mothers confirmed that everything is clear, they were asked one last time if they wanted to participate in the study. The data collectors circled either 'yes' or 'no' depending on which option was chosen, and the interview was only conducted if the data collector was instructed to circle the response 'yes'”.* We have included the above detail under method section in “Ethical consideration” (Revised manuscript, line #292-297).

**Please review your reference list to ensure that it is complete and correct. If you have cited papers that have been retracted, please include the rationale for doing so in the manuscript text, or remove these references and replace them with relevant current references. Any changes to the reference list should be mentioned in the rebuttal letter that accompanies your revised manuscript. If you need to cite a retracted article, indicate the article’s retracted status in the References list and also include a citation and full reference for the retraction notice.**

**Response:** We've updated the reference list to make it complete and accurate. We updated the references to conform to the International Committee of Medical Journal Editors' reference format (ICMJE). The following is a list of updated references to ensure that it is complete and accurate, as required by the journal.

Reference number 2, 4, 5, 7, 8, 10, 12, 14, 15, 17-27, and 31-46: According to ICMJE citation style, it is an online paper with a DOI. As a result, we have included the entire DOI next to the publication date in the revised manuscript.

Reference number 2, 4, 7, 8, 15, 21, 23, 25, 26, 31, 32, 35, 37-39, 41-43, and 45: In the previous format the journal titles were presented in its full form, however according to the PLOS ONE guide journal titles should be abbreviated according to NLM Catalog of NCBI Journals. Accordingly we have revised the journals title in the revised manuscript.

Reference number 3, 6, 9 and 30: The source is a website, but the accessed date and availability were missing in the previous format. We have made the necessary changes.

Reference number 1, 2, 4, 5, 7, 8, 10-29, 31-42, and 44-46: In the previous format the publication date was presented incorrectly. Now we have revised it by including the publication date in the right place next to abbreviated journal title.

Reference number 1, 2, 4, 7, 8, 18, 20, 24, 32, 39 and 46: In the previous format the authors list were not presented according to the reference style used by PLOS ONE journal. Now we have revised it by listing the authors surname first followed by their first and middle initials, in the revised manuscript.

**REPLY TO REVIEWERS’ COMMENTS**

**Comments from reviewer # 1**

**Reviewer #1: the theoretical framework of matched sampling and that of logistic regression may be expanded a little more. also, there are typos which need to be addressed, for example, lines 57-58 need to be re-framed as "The devastating effect of coronavirus disease 2019 pandemic on maternity services should be prevented.**

**Response:** We thank the reviewer for useful comment and suggestion. We agree with the reviewer that the theoretical framework of matched sampling and logistic regression is expanded a little more in our manuscript. Now, we have revised it to make it short and precise (Revised manuscript, line #252-258, line#265-267, line#270-271). In addition, we have corrected the typos error (Revised manuscript, line #57).

**Comments from reviewer # 2**

**Reviewer #2: Dear author, thank you for submitting this work as home birth is common problem in low-income countries, and lead to adverse health outcomes of both the mothers and their babies. Overall, you did great but there are points that need revision. Here are my comments and questions.**

**Response:** Thank you very much for your nice wording.

**General comment on method: - case control study design is preferred study design in case of rare disease (outcome of interest) while home delivery is very common scenario particularly in low-income countries like Ethiopia. So why the author prefers this study designs over others e.g., correctional study design. I suggest the author at least to discuss it briefly.**

**Response:** We thank the reviewer for raising critical question. As the reviewer mentioned case control is preferred study design for rare disease, and home delivery is very common practice particularly in our country context. However, as we have mentioned in the background section, the prevalence of home delivery is well studied and known in our setting, even one can get about the exact figure of home delivery from the district health office. However, there are limited studies which investigate the factors that affect home birth. Because of this reasons our study objective was to identify multiple factors that affect home birth and Case-control study is an efficient research method for investigating risk factors of home birth. We believe that case control is appropriate for the identified research problem and the best option for answering our research question.

**1. methods section: what about mothers who might gave birth at private health facilities?? Did you included or excluded from the study? Please explain it.**

**Response:** Thank you for raising an important point here. Before conducting our study during proposal phase, we have considered to include mothers who gave birth at private health facilities to avoid selection bias. However, despite there are some private clinics (medium and primary) at wondo Genet, none of them provide delivery service. That is why we didn’t consider it.

**2. methods section: in your sampling procedure, you stated that you selected three health facilities out of five health facilities using simple random sampling technique, and then take sample of mothers from these HF. What was the sampling method used to select study participants from the three HFs? You didn’t explain anything about muti stage sampling kindly give explanation about it.**

**Response:** We apologize for lack of clarity in our first manuscript regarding the sampling method we have used after selecting three health facilities out of five. First we have selected three health facilities out of five using simple random sampling technique. Then, the sample size was proportionally allocated based on the client flow for immunization and post natal services at each of the three health facilities in the year proceeding the study period. Mothers who give birth during last 12 months and visited the selected health facilities were classified as case and control based on where they delivered. Because of difficulty for preparing an exact sample frame of case and control based on health facility reports of immunization and post natal care (PNC) service, and the estimated number of cases and controls for data collection period (one month) were not large enough, we used the consecutive sampling method. Based on the give comment, we have included a sentence that describes the sampling method we used to select study participants from three health facilities (Revised manuscript, line# 199).

**3. methods section: As you know there are three control selection methods in Case-control studies’ namely: Survivor sampling, Case-base or case-cohort sampling and Risk set sampling. kindly give the control selection method you used.**

**Response:** we would like to thank the reviewer for the detail comment. As correctly mentioned by the reviewer there are three control selection methods. Among these methods we have used survivor sampling or cumulative density sampling because controls are selected from those who remain free of the outcome variable (home delivery) or from those who give birth at health facility during 12 months before the data collection period. It is not risk set sampling or incident sampling technique because in this sampling method the controls are sampled at the time of the occurrence of a case, and we usually use this method when the cases are incident cases. But our study used prevalent case since we considered mothers who had given birth in the last 12 months before the data collection period. In addition, it is not also case based or case cohort sampling because this type of sampling only works with a previously defined cohort where controls are selected from the population at risk at the beginning of the follow-up period in the cohort study.

**4. discussion section: your conclusion and recommendation should be based on your finding. For example, in the conclusion part you stated that “long distances coupled with poor road systems play a significant role in most 555 rural areas…” But you didn.t used road system variable in your analysis. Additionally, you conclude that “Gender inequalities denying women’s independent decision making, education, and freedom of movement restrict use of maternal health services including facility delivery”. Please conclude based on what you found in your statistical analysis.**

**Response:** We thank the Reviewer for the critical review and the useful suggestions. Comment is accepted and correction is made (Revised manuscript, line #574-587).

**5. discussion section: kindly give the strength of this study**

**Response:** Thank you for your suggestion; accordingly we have incorporated your suggestion (Revised manuscript, line #558-561).
