# Supplementary material for: Predictors of homebirth amidst COVID-19 pandemic among women attending health facilities in Wondo Genet, Sidama Region, Ethiopia: A case control study
Source: PLoS One. 2023 May 2;18(5):e0283547. doi: 10.1371/journal.pone.0283547 (PMC10153687; doi:10.1371/journal.pone.0283547)
Supplement: Attachment — Submitted filename: Response to Reviewers.docx [file pone.0283547.s005.docx]

Yilkal Simachew

Lecturer and Researcher

Hawassa University, Ethiopia

[joemakalister123@gmail.com](mailto:joemakalister123@gmail.com)

Dr. Shabnam Iezadi

Academic Editor

*Journal of PLOS ONE*

[em@editorialmanager.com](mailto:em@editorialmanager.com)	March 07, 2023

**Subject:** Revision and resubmission of manuscript (PONE-D-22-17306)

Dear Dr. Shabnam Iezadi

Thank you for giving us the opportunity to revise and resubmit the manuscript “Predictors of homebirth amidst COVID-19 pandemic among women attending health facilities in Wondo Genet, Sidama Region, Ethiopia: a case control study”, for publication in the *journal of PLOS ONE.* We wish to express our appreciation to the editor and the anonymous reviewers for your in-depth comments, suggestion, and corrections, which have greatly improved the manuscript. We have carefully considered all suggestions and concerns and tried our best to address every one of them. The changes are marked in the revised manuscript.

We have included the Editor's Comments immediately after this letter and responded to them individually, indicating exactly how we addressed each concern and describing the changes we have made. Please note that Editor's comments are shown in **bold** type and our responses in plain type. Changes to the manuscript that have been made are marked in blue color in the revised version. The removal of text at specific locations is highlighted in ~~red~~ color in the revised manuscript.

We thank you for your continued interest in our research.

Sincerely,

Yilkal Simachew

Lecturer and Researcher at Hawassa University, in Ethiopia

**REPLY TO THE ACADEMIC EDITOR’S COMMENTS**

**Comment 1: Dear authors, Thank you for making the necessary revisions. There are just a few points that need to be addressed before we can make the final decision. 1-There are still some typo errors, line 286 (76.6% %) for example. Please, kindly review the whole manuscript and edit all typos.**

Response: Thank you for advising us to review the whole manuscript and edit the typos errors. Now, we have revised and edited the typos errors throughout the manuscript. The changes that have been made are found in the revised manuscript with track changes.

**Comment 2: In line 184 in sample size and sampling procedures section, reference number 61 does not exist in the reference list. Please check this out and kindly review the references for any mismatch.**

Response: Thank you for pointing this out. To calculate the sample size, we took those parameters from the study conducted in Northern Ethiopia with reference number 44 (*Tsegay R, Aregay A, Kidanu K, Alemayehu M, Yohannes G. Determinant factors of home delivery among women in Northern Ethiopia: a case control study. BMC Public Health. 2017 Apr 4;17(1):289. doi: 10.1186/s12889-017-4159-1*). Now, we have revised it accordingly (Revised manuscript, line #187).

**Comment 3: Please, add a statement to the text to indicate that "despite there are some private clinics (medium and primary) at wondo Genet, none of them provide delivery service". Although you have explained this in your response letter, there is no change in the body of the manuscript to show your explanation.**

Response: We have added the above statement in the revised manuscript under the method and materials section in the study area (Revised manuscript, line #164-165).

**Comment 4: Please, add a statement to the text (method section) about the control selection sampling method (survivor sampling or cumulative density sampling). Although you have explained this in your response letter, there is no change in the body of the manuscript to show your explanation.**

Response: We have included a statement about the control selection sampling method under the method section in the sample size and sampling procedure subsection (Revised manuscript, line #198-200).
